# Association of Intensive Care Unit Patient Load and Demand With Mortality Rates in US Department of Veterans Affairs Hospitals During the COVID-19 Pandemic

**DOI:** 10.1001/jamanetworkopen.2020.34266

**Published:** 2021-01-19

**Authors:** Dawn M. Bravata, Anthony J. Perkins, Laura J. Myers, Greg Arling, Ying Zhang, Alan J. Zillich, Lindsey Reese, Andrew Dysangco, Rajiv Agarwal, Jennifer Myers, Charles Austin, Ali Sexson, Samuel J. Leonard, Sharmistha Dev, Salomeh Keyhani

**Affiliations:** 1 Precision Monitoring to Transform Care Quality Enhancement Research Initiative, Health Services Research and Development, Department of Veterans Affairs, Indianapolis, Indiana; 2 Health Services Research and Development Center for Health Information and Communication, Richard L. Roudebush VA Medical Center, Department of Veterans Affairs, Indianapolis, Indiana; 3 Medicine Service, Richard L. Roudebush VA Medical Center, Indianapolis, Indiana; 4 Department of Medicine, Indiana University School of Medicine, Indianapolis; 5 Department of Neurology, Indiana University School of Medicine, Indianapolis; 6 William M. Tierney Center for Health Services Research, Regenstrief Institute, Indianapolis, Indiana; 7 Department of Biostatistics, Indiana University School of Medicine, Indianapolis; 8 School of Nursing, Purdue University, West Lafayette, Indiana; 9 Department of Biostatistics, College of Public Health, University of Nebraska Medical Center, Omaha; 10 Department of Pharmacy Practice, College of Pharmacy, Purdue University, West Lafayette, Indiana; 11 Northern California Institute for Research and Education, San Francisco; 12 San Francisco VA Medical Center, San Francisco, California; 13 Division of General Internal Medicine, Department of Medicine, University of California, San Francisco

## Abstract

**Importance:**

Although strain on hospital capacity has been associated with increased mortality in nonpandemic settings, studies are needed to examine the association between coronavirus disease 2019 (COVID-19) critical care capacity and mortality.

**Objective:**

To examine whether COVID-19 mortality was associated with COVID-19 intensive care unit (ICU) strain.

**Design, Setting, and Participants:**

This cohort study was conducted among veterans with COVID-19, as confirmed by polymerase chain reaction or antigen testing in the laboratory from March through August 2020, cared for at any Department of Veterans Affairs (VA) hospital with 10 or more patients with COVID-19 in the ICU. The follow-up period was through November 2020. Data were analyzed from March to November 2020.

**Exposures:**

Receiving treatment for COVID-19 in the ICU during a period of increased COVID-19 ICU load, with load defined as mean number of patients with COVID-19 in the ICU during the patient’s hospital stay divided by the number of ICU beds at that facility, or increased COVID-19 ICU demand, with demand defined as mean number of patients with COVID-19 in the ICU during the patient’s stay divided by the maximum number of patients with COVID-19 in the ICU.

**Main Outcomes and Measures:**

All-cause mortality was recorded through 30 days after discharge from the hospital.

**Results:**

Among 8516 patients with COVID-19 admitted to 88 VA hospitals, 8014 (94.1%) were men and mean (SD) age was 67.9 (14.2) years. Mortality varied over time, with 218 of 954 patients (22.9%) dying in March, 399 of 1594 patients (25.0%) dying in April, 143 of 920 patients (15.5%) dying in May, 179 of 1314 patients (13.6%) dying in June, 297 of 2373 patients (12.5%) dying in July, and 174 of 1361 (12.8%) patients dying in August (*P* < .001). Patients with COVID-19 who were treated in the ICU during periods of increased COVID-19 ICU demand had increased risk of mortality compared with patients treated during periods of low COVID-19 ICU demand (ie, demand of ≤25%); the adjusted hazard ratio for all-cause mortality was 0.99 (95% CI, 0.81-1.22; *P* = .93) for patients treated when COVID-19 ICU demand was more than 25% to 50%, 1.19 (95% CI, 0.95-1.48; *P* = .13) when COVID-19 ICU demand was more than 50% to 75%, and 1.94 (95% CI, 1.46-2.59; *P* < .001) when COVID-19 ICU demand was more than 75% to 100%. No association between COVID-19 ICU demand and mortality was observed for patients with COVID-19 not in the ICU. The association between COVID-19 ICU load and mortality was not consistent over time (ie, early vs late in the pandemic).

**Conclusions and Relevance:**

This cohort study found that although facilities augmented ICU capacity during the pandemic, strains on critical care capacity were associated with increased COVID-19 ICU mortality. Tracking COVID-19 ICU demand may be useful to hospital administrators and health officials as they coordinate COVID-19 admissions across hospitals to optimize outcomes for patients with this illness.

## Key Points

**Question:**

Is greater coronavirus disease 2019 (COVID-19) intensive care unit (ICU) strain associated with increased COVID-19 mortality?

**Findings:**

In this cohort study of 8516 patients with COVID-19 admitted to 88 US Veterans Affairs hospitals, strains on critical care capacity were associated with increased COVID-19 mortality. Among patients with COVID-19, those treated in the ICU during periods of peak COVID-19 ICU demand had a nearly 2-fold increased risk of mortality compared with those treated during periods of low demand.

**Meaning:**

These findings suggest that public health officials and hospital administrators should consider interventions that reduce COVID-19 ICU demand to improve survival among patients with COVID-19 in the ICU.

## Teaser

This cohort study examines the association of patient caseload and demand with mortality among patients with coronavirus disease 2019 (COVID-19) in US Veterans Affairs (VA) intensive care units.

## Introduction

Health policy interventions (eg, social distancing) implemented to avoid overloading health care systems have been associated with reduced coronavirus disease 2019 (COVID-19) hospitalization rates.^1,2^ Hospital capacity strain resulting from increased patient volume or disease severity is associated with increased mortality in nonpandemic settings.^3^ The association between COVID-19 critical care strain and mortality has not been examined. The objective of this study was to examine the associations between 2 measures of intensive care unit (ICU) strain and mortality among patients with COVID-19 who were admitted to a Department of Veterans Affairs (VA) facility.

## Methods

This cohort study was approved by the Indiana University School of Medicine institutional review board and the VA research and development committee at Richard L. Roudebush VA Medical Center. This was an observational study without any direct patient contact and considered to be of minimal risk; therefore, a waiver of informed consent was obtained. This study adheres to the Strengthening the Reporting of Observational Studies in Epidemiology (STROBE) reporting guideline.

This observational cohort study included patients with polymerase chain reaction or antigen test results positive for severe acute respiratory syndrome coronavirus 2 (SARS-CoV-2) from March 1 to August 31, 2020. We included patients admitted to a VA hospital that had 10 or more patients with COVID-19 in the ICU during the study period. We excluded patients whose SARS-CoV-2 test date was more than 2 days after admission. Follow-up extended through November 1, 2020.

Data from the VA Corporate Data Warehouse (CDW), including inpatient and outpatient data files from the 2 years before the patient’s COVID-19 diagnosis through the day of admission, were used to identify comorbid conditions, self-reported race/ethnicity, health care use, procedures received, vital signs, and laboratory data.^4,5^ Death dates were obtained from the CDW, VA Vital Status File, and patient’s electronic health record.^6^ The modified Acute Physiology, Age, Chronic Health Evaluation (APACHE) III score^7^ was calculated at the time of hospital admission as a measure of illness severity. Number of ICU beds was obtained from the VA Bed Management Solution initiative. The number of ICU beds per facility described prepandemic ICU capacity, not any augmented capacity that may have been implemented during the COVID-19 pandemic. Facility complexity described the level of services provided at a VA facility, categorized as 1a, 1b, 1c, 2, or 3, with level 1a being the most complex and level 3 being the least complex. Facility complexity included ICU level, operative complexity level, patient clinical classification, teaching status characteristics, amount of research funding, complex clinical programs provided (eg, invasive catheterization laboratory, neurosurgery, and transplant), rurality, care provided in the community, and mental health programs provided. The primary outcome was all-cause mortality. Patients were followed up for 30 days after discharge.

### Measures of COVID-19 ICU Strain

**Figure 1.**
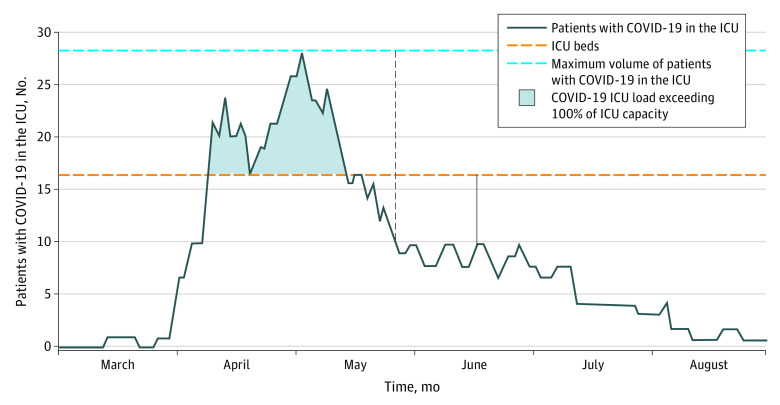
Coronavirus Disease 2019 (COVID-19) Intensive Care Unit (ICU) Load and Demand at an Example Facility Black solid vertical line with arrows indicates the numbers at 1 time point used to calculate COVID-19 ICU load, defined as the mean number of patients with COVID-19 in the ICU during a patient’s hospital stay divided by the number of ICU beds. Black dotted vertical line indicates the numbers at 1 time point used to calculate COVID-19 demand, defined as the mean number of patients with COVID-19 in the ICU during a patient’s hospital stay divided by the maximum number of patients with COVID-19 in the ICU during the study period. The results suggest that the risk of mortality would be highest if a COVID-19 patient’s stay was during the peak of ICU demand and if ICU caseload approached or exceeded ICU bed capacity.

This study included 2 measures of COVID-19 critical care strain: ICU load and ICU demand; Figure 1 displays how these measures vary over time within an example facility. The COVID-19 ICU load described how the caseload of patients with COVID-19 in the ICU compared with the typical ICU bed count at each facility. The COVID-19 ICU load was calculated at the patient level as the mean number of patients with COVID-19 in the ICU during the patient’s hospital stay divided by the number of ICU beds at that facility. The number of ICU beds at each facility was a fixed number. The COVID-19 ICU load included only the number of patients with COVID-19 in the ICU, excluding patients with other critical illnesses. The COVID-19 ICU load ranged from 0% to greater than 100%; it exceeded 100% if the hospital increased critical care bed capacity during the pandemic (eg, by converting a sleep laboratory into an ICU) and if those beds were occupied by patients with COVID-19.

The population of patients with COVID-19 in the ICU varied over time, with peak prevalence rates occurring early (eg, March) at some hospitals and later (eg, July) at other hospitals. The COVID-19 ICU demand described the caseload of patients with COVID-19 in the ICU when a patient was treated compared with peak COVID-19 ICU caseload. It was calculated at the patient level as the mean number of patients with COVID-19 in the ICU during the patient’s stay divided by the maximum number of patients with COVID-19 in the ICU at that facility during the study period. The COVID-19 ICU demand ranged from 0% to 100%.

For example, if a hospital had 60 ICU beds before the pandemic and the mean number of patients with COVID-19 in the ICU during a patient’s stay was 20, then the COVID-19 ICU load would be 20 divided by 60, or 33%. If at that same facility the peak surge included 20 patients with COVID-19 in the ICU and a patient was treated during the period when the mean of number patients with COVID-19 in the ICU was 20, then the COVID-19 ICU demand would be 20 divided by 20, or 100%.

### Statistical Analysis

We described differences over time in baseline characteristics and mortality among inpatients with COVID-19 using χ^2^ and Wilcoxon rank sum tests. We used Cox proportional hazard models to analyze the time in days from admission to death, either in the hospital or within 30 days after discharge, among patients who were admitted to the hospital (overall, in the general ward, and in the ICU). Patients who were still in the hospital or out of the hospital and alive at 30 days after discharge were treated as censored observations. We included a random effect for the facility to account for the correlation of mortality among patients in the same hospital. Analyses were performed using SAS Enterprise Guide statistical software version 7.11 (SAS Institute). *P* values were 2-sided, and statistical significance was set at *P* < .05. Data were analyzed from March to November 2020.

## Results

**Figure 2.**
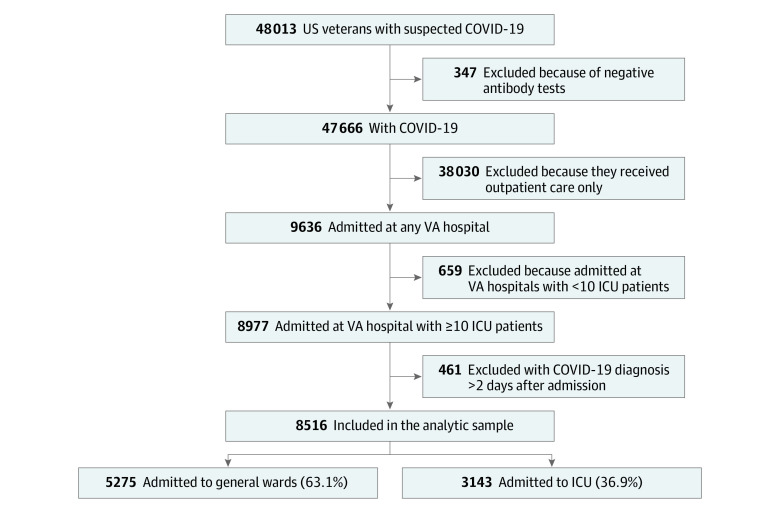
Patient Flow Diagram The figure displays the relative proportion of veterans with coronavirus disease 2019 (COVID-19) who were cared for in the general ward and intensive care unit (ICU).

The cohort included 8516 patients with COVID-19 admitted to 88 VA hospitals (Figure 2); 8014 (94.1%) were men, and mean (SD) age was 67.9 (14.2) years. The observed mortality was 218 of 954 patients (22.9%) in March. It increased to 399 of 1594 patients (25.0%) in April, then decreased to 143 of 920 patients (15.5%) in May, leveling off at 179 of 1314 patients (13.6%) in June, 297 of 2373 patients (12.5%) in July, and 174 of 1361 patients (12.8%) in August (*P* < .001) (Table 1). The proportion of patients ages 75 years or older fluctuated over time; 223 patients (23.4%) were in this age group in March, increasing to 534 patients (33.5%) in April and 311 patients (33.8%) in May, decreasing to 333 patients (25.3%) in June and 637 patients (26.8%) in July, and then increasing again to 435 patients (32.0%) in August (*P* < .001). The proportion of patients receiving care on general wards increased after the earliest months of the pandemic (March: 527 patients [55.2%]; April: 965 patients [60.5%]; May: 546 patients [59.3%]; June: 829 patients [63.1%]; July: 1603 patients [67.6%]; and August: 903 patients [66.3%]; *P* < .001). The COVID-19 ICU load and demand changed over time. The proportion of patients with COVID-19 treated during periods of low COVID-19 ICU load (ie, ≤25%) increased over time, with 487 patients (51.0%) in March, 952 patients (59.7%) in April, 785 patients (85.3%) in May, 1170 patients (89.0%) in June, 1923 patients (81.0%) in July, and 1250 patients (91.8%) in August (*P* < .001). Similarly, the proportion of patients with COVID-19 treated during periods of peak COVID-19 ICU load (ie, >100%) decreased from 60 patients (6.3%) in March to 18 patients (1.1%) in April, to 0 patients in May through August. The proportion of patients with COVID-19 treated during periods of high COVID-19 ICU demand (ie, >75%) decreased during the first 3 months of the pandemic, with 233 patients (24.4%) in March, 322 patients (20.2%) in April, and 44 patients (4.8%) in May; increased to 142 patients (10.8%) in June and 413 patients (17.4%) in July; then decreased to 79 patients (5.8%) in August (*P* < .001) (Table 1).

**Table 1.** Baseline Characteristics and Mortality Over Time

Characteristic, No. (%)	Patients with COVID-19 by test date, No. (%)						*P* value
	March (n = 954)	April (n = 1594)	May (n = 920)	June (n = 1314)	July (n = 2373)	August (n = 1361)	
Age, y							
<65	412 (43.2)	499 (31.3)	275 (29.9)	564 (42.9)	952 (40.1)	472 (34.7)	<.001
65-74	319 (33.4)	561 (35.2)	334 (36.3)	417 (31.7)	784 (33.0)	454 (33.4)	
75-84	147 (15.4)	306 (19.2)	173 (18.8)	207 (15.8)	410 (17.3)	269 (19.8)	
≥85	76 (8.0)	228 (14.3)	138 (15.0)	126 (9.6)	227 (9.6)	166 (12.2)	
Men	907 (95.1)	1518 (95.2)	872 (94.8)	1216 (92.5)	2228 (93.9)	1273 (93.5)	.03
Race/ethnicity							
African American	591 (61.9)	822 (51.6)	402 (43.7)	504 (38.4)	887 (37.4)	458 (33.7)	<.001
Other or unknown^a^	54 (5.7)	88 (5.5)	62 (6.7)	115 (8.8)	199 (8.4)	116 (8.5)	
White	309 (32.4)	684 (42.9)	456 (49.6)	695 (52.9)	1287 (54.2)	787 (57.8)	
Primary care visit in prior 2 y	912 (95.6)	1479 (92.8)	834 (90.7)	1254 (95.4)	2243 (94.5)	1256 (92.3)	<.001
COVID-19 ICU load^b^							
≤25%	487 (51.0)	952 (59.7)	785 (85.3)	1170 (89.0)	1923 (81.0)	1250 (91.8)	<.001
>25% to 50%	141 (14.8)	305 (19.1)	77 (8.4)	126 (9.6)	443 (18.7)	106 (7.8)	
>50% to 75%	178 (18.7)	181 (11.4)	53 (5.8)	17 (1.3)	7 (0.3)	5 (0.4)	
>75% to 100%	88 (9.2)	138 (8.7)	5 (0.5)	1 (0.1)	0	0	
>100%	60 (6.3)	18 (1.1)	0	0	0	0	
COVID-19 ICU demand^c^							
≤25%	241 (25.3)	336 (21.1)	381 (41.4)	472 (35.9)	516 (21.7)	542 (39.8)	<.001
>25% to 50%	219 (23.0)	466 (29.2)	307 (33.4)	434 (33.0)	772 (32.5)	498 (36.6)	
>50% to 75%	261 (27.4)	470 (29.5)	188 (20.4)	266 (20.2)	672 (28.3)	242 (17.8)	
>75%	233 (24.4)	322 (20.2)	44 (4.8)	142 (10.8)	413 (17.4)	79 (5.8)	
Hypertension	727 (76.2)	1272 (79.8)	706 (76.7)	970 (73.8)	1774 (74.8)	1023 (75.2)	.002
Atrial fibrillation	127 (13.3)	302 (18.9)	174 (18.9)	191 (14.5)	362 (15.3)	231 (17.0)	<.001
Smoking history							
Never	336 (35.2)	449 (28.2)	248 (27.0)	480 (36.5)	780 (32.9)	442 (32.5)	<.001
Current	89 (9.3)	213 (13.4)	128 (13.9)	164 (12.5)	346 (14.6)	204 (15.0)	
Former	420 (44.0)	661 (41.5)	364 (39.6)	546 (41.6)	1006 (42.4)	532 (39.1)	
Unknown	109 (11.4)	271 (17.0)	180 (19.6)	124 (9.4)	241 (10.2)	183 (13.4)	
BMI							
<25 (reference)	193 (20.2)	451 (28.3)	274 (29.8)	307 (23.4)	555 (23.4)	328 (24.1)	<.001
25-29	271 (28.4)	481 (30.2)	278 (30.2)	405 (30.8)	702 (29.6)	416 (30.6)	
30-34	260 (27.3)	339 (21.3)	194 (21.1)	315 (24.0)	573 (24.1)	323 (23.7)	
≥35	218 (22.9)	300 (18.8)	146 (15.9)	267 (20.3)	513 (21.6)	265 (19.5)	
Missing data	12 (1.3)	23 (1.4)	28 (3.0)	20 (1.5)	30 (1.3)	29 (2.1)	
Place of treatment							
General ward	527 (55.2)	965 (60.5)	546 (59.3)	829 (63.1)	1603 (67.6)	903 (66.3)	<.001
ICU							
No ventilation	195 (20.4)	367 (23.0)	251 (27.3)	346 (26.3)	536 (22.6)	341 (25.1)	
Mechanical ventilation	232 (24.3)	262 (16.4)	123 (13.4)	139 (10.6)	234 (9.9)	117 (8.6)	
APACHE score, median (IQR)^d^	12 (6-21)	13 (6-22)	12 (5-20)	10 (4-17)	10 (4-19)	10 (4-18)	<.001
Charlson comorbidity index, median (IQR)^e^	3 (1-6)	3 (1-6)	3 (1-6)	2 (1-5)	3 (1-5)	3 (1-5)	<.001
Died	218 (22.9)	399 (25.0)	143 (15.5)	179 (13.6)	297 (12.5)	174 (12.8)	<.001

Abbreviations: APACHE, Acute Physiology, Age, Chronic Health Evaluation; BMI, body mass index (calculated as weight in kilograms divided by height in meters squared); COVID-19, coronavirus disease 2019; ICU, intensive care unit; IQR, interquartile range.

^a^ Other includes Hispanic or Latino, Asian, Native Hawaiian or other Pacific Islander, American Indian or Alaskan Native, or other race/ethnicity.

^b^ Calculated as mean No. of patients with COVID-19 in the ICU during stay divided by No. of ICU beds.

^c^ Calculated as mean No. of patients with COVID-19 in the ICU during stay divided by maximum No. of patients with COVID-19 in the ICU.

^d^ A physiologic measure in which increasing scores indicate worse health. This score includes laboratory data (eg, white blood cell count) as well as vital signs (eg, oxygenation and blood pressure).

^e^ A measure of medical comorbidity in which increasing values indicate a greater comorbidity burden.

Patients with COVID-19 in the ICU treated during high COVID-19 ICU strain had increased risk of mortality. Table 2 provides the unadjusted results, and Table 3 displays the adjusted hazard ratios (HRs). Compared with patients with COVID-19 in the ICU treated during periods of low COVID-19 ICU load (ie, ≤25%), the adjusted HR for all-cause mortality was 1.10 (95% CI, 0.88-1.37) for patients treated during periods when COVID-19 ICU load was greater than 25% to 50%, 1.15 (95% CI, 0.81-1.64) when COVID-19 ICU load was greater than 50% to 75%, 1.67 (95% CI, 1.08-2.60) when COVID-19 ICU load was greater than 75% to 100%, and 2.35 (95% CI, 1.25-4.39) when COVID-19 ICU load was 100% or more (*P* = .049). The association between COVID-19 ICU load and mortality among patients treated in the general ward was statistically significant but did not form a monotonic gradient; the adjusted HR for all-cause mortality was 1.30 (95% CI, 0.92-1.84) for patients treated during periods when COVID-19 ICU load was greater than 25% to 50%, 0.74 (95% CI, 0.42-1.32) when COVID-19 ICU load was greater than 50% to 75%, 1.90 (95% CI, 0.98-3.65) when COVID-19 ICU load was greater than 75% to 100%, and 1.14 (95% CI, 0.29-4.49) when COVID-19 ICU load was 100% or more (*P* = .04).

**Table 2.** Unadjusted Mortality by COVID-19 ICU Strain Metrics

ICU strain at patient level	COVID-19 mortality through 30 d postdischarge, No./total No. (%)		
	Overall	General ward only	ICU
**COVID-19 ICU load**^a^			
≤25%	950/6567 (14.5)	313/4303 (7.3)	637/2264 (28.1)
>25% to 50%	259/1198 (21.6)	75/662 (11.3)	184/536 (34.3)
>50% to 75%	96/441 (21.8)	22/233 (9.4)	74/208 (35.6)
>75% to 100%	79/232 (34.0)	28/132 (21.2)	51/100 (51.0)
>100%	26/78 (33.3)	3/43 (7.0)	23/35 (65.7)
**COVID-19 ICU demand**^b^			
≤25%	281/2488 (11.3)	116/1815 (6.4)	165/673 (24.5)
>25% to 50%	429/2696 (15.9)	145/1683 (8.6)	284/1013 (28.0)
>50% to 75%	443/2099 (21.1)	106/1107 (9.6)	337/992 (34.0)
>75%	257/1233 (20.8)	74/768 (9.6)	183/465 (39.4)

Abbreviations: COVID-19, coronavirus disease 2019; ICU, intensive care unit.

^a^ Calculated as No. patients with COVID-19 in the ICU during stay divided by No. of ICU beds.

^b^ Calculated as mean No. of patients with COVID-19 in the ICU during stay divided by maximum No. of patients with COVID-19 in the ICU.

**Table 3.** Proportional Hazard Results From Admission to 30 Days Postdischarge or Death

Characteristic	Overall		General ward only		ICU	
	Adjusted HR (95% CI)	*P* value	Adjusted HR (95% CI)	*P* value	Adjusted HR (95% CI)	*P* value
Age, y						
<65	1 [Reference]	<.001	1 [Reference]	<.001	1 [Reference]	<.001
65-74	2.10 (1.75-2.52)		2.35 (1.55-3.57)		2.08 (1.70-2.55)	
75-84	3.04 (2.50-3.69)		4.79 (3.15-7.29)		2.73 (2.18-3.42)	
≥85	6.85 (5.59-8.39)		11.15 (7.33-16.97)		5.39 (4.20-6.92)	
Women	0.86 (0.60-1.25)	.44	0.68 (0.32-1.46)	.32	0.97 (0.63-1.48)	.88
Race/ethnicity						
African American	0.77 (0.68-0.87)	<.001	0.61 (0.48-0.77)	<.001	0.86 (0.74-1.00)	.11
Other or unknown^a^	1.01 (0.81-1.26)		0.93 (0.64-1.36)		1.02 (0.78-1.34)	
White	1 [Reference]		1 [Reference]		1 [Reference]	
Primary care within prior 2 y	0.86 (0.68-1.09)	.20	0.78 (0.54-1.14)	.20	0.93 (0.69-1.26)	.63
COVID-19 ICU load^b^						
≤25%	1 [Reference]	.01	1 [Reference]	.04	1 [Reference]	.049
>25% to 50%	1.19 (0.99-1.43)		1.30 (0.92-1.84)		1.10 (0.88-1.37)	
>50% to 75%	1.03 (0.77-1.40)		0.74 (0.42-1.32)		1.15 (0.81-1.64)	
>75% to 100%	1.63 (1.13-2.35)		1.90 (0.98-3.65)		1.67 (1.08-2.60)	
>100%	2.03 (1.16-3.56)		1.14 (0.29-4.49)		2.35 (1.25-4.39)	
COVID-19 ICU demand^c^						
≤25%	1 [Reference]	<.001	1 [Reference]	.09	1 [Reference]	<.001
>25% to 50%	1.11 (0.94-1.31)		1.41 (1.07-1.84)		0.99 (0.81-1.22)	
>50% to 75%	1.25 (1.05-1.49)		1.30 (0.95-1.79)		1.19 (0.95-1.48)	
>75%	1.67 (1.33-2.11)		1.29 (0.85-1.97)		1.94 (1.46-2.59)	
Hypertension	0.94 (0.80-1.11)	.46	0.92 (0.68-1.24)	.55	0.94 (0.77-1.14)	.51
Atrial fibrillation	1.00 (0.88-1.15)	.95	1.01 (0.81-1.27)	.93	1.00 (0.84-1.18)	.96
History of smoking						
Never	1 [Reference]	<.001	1 [Reference]	.001	1 [Reference]	<.001
Current	0.70 (0.56-0.87)		0.73 (0.47-1.14)		0.74 (0.57-0.96)	
Former	0.93 (0.81-1.06)		0.98 (0.77-1.26)		0.92 (0.78-1.08)	
Unknown	1.55 (1.30-1.84)		1.59 (1.19-2.12)		1.59 (1.27-1.98)	
BMI						
<25	1 [Reference]	.001	1 [Reference]	.01	1 [Reference]	.12
25-29	0.83 (0.72-0.95)		0.75 (0.59-0.94)		0.88 (0.74-1.05)	
30-34	0.80 (0.68-0.94)		0.72 (0.53-0.97)		0.84 (0.69-1.02)	
≥35	0.94 (0.79-1.11)		0.96 (0.68-1.36)		0.98 (0.79-1.20)	
Missing data	1.38 (0.97-1.98)		1.65 (0.92-2.93)		1.39 (0.87-2.22)	
Place of treatment						
General ward	1 [Reference]	<.001	NA	NA	NA	<.001
ICU						
No ventilation	1.93 (1.66-2.24)		NA		1 [Reference]	
Mechanical ventilation	7.40 (6.47-8.48)		NA		3.93 (3.39-4.55)	
APACHE score^d^	1.02 (1.02-1.03)	<.001	1.04 (1.03-1.05)	<.001	1.02 (1.01-1.02)	<.001
Charlson comorbidity score^e^	1.05 (1.03-1.06)	<.001	1.09 (1.06-1.12)	<.001	1.02 (1.00-1.05)	.03
Month of COVID diagnosis						
March	1.46 (1.15-1.84)	<.001	1.43 (0.92-2.22)	.004	1.50 (1.13-2.00)	.009
April	1.47 (1.20-1.79)		1.48 (1.04-2.10)		1.52 (1.19-1.96)	
May	1 [Reference]		1 [Reference]		1 [Reference]	
June	1.15 (0.91-1.45)		1.11 (0.74-1.68)		1.22 (0.91-1.62)	
July	1.05 (0.85-1.30)		0.83 (0.57-1.20)		1.23 (0.94-1.61)	
August	1.23 (0.97-1.55)		0.84 (0.56-1.26)		1.50 (1.13-2.00)	
Facility complexity^f^						
1a	1.05 (0.67-1.66)	.08	1.41 (0.54-3.68)	.80	0.88 (0.52-1.50)	.08
1b	0.94 (0.59-1.51)		1.29 (0.48-3.47)		0.82 (0.47-1.41)	
1c	1.29 (0.81-2.07)		1.50 (0.56-4.04)		1.17 (0.68-2.02)	
2	1 [Reference]		1 [Reference]		1 [Reference]	

Abbreviations: APACHE, Acute Physiology, Age, Chronic Health Evaluation; BMI, body mass index (calculated as weight in kilograms divided by height in meters squared); COVID-19, coronavirus disease 2019; HR, hazard ratio; ICU, intensive care unit; NA, not applicable.

^a^ The other race/ethnicity category includes Hispanic or Latino, Asian, Native Hawaiian or other Pacific Islander, American Indian or Alaskan Native, or other race/ethnicity.

^b^ Calculated as No. patients with COVID-19 in the ICU during stay divided by No. of ICU beds.

^c^ Calculated as mean No. of patients with COVID-19 in the ICU during stay divided by maximum No. of patients with COVID-19 in the ICU.

^d^ A physiologic measure in which increasing scores indicate worse health. This score includes laboratory data (eg, white blood cell count) as well as vital signs (eg, oxygenation and blood pressure).

^e^ A measure of medical comorbidity in which increasing values indicate a greater comorbidity burden.

^f^ The complexity of the services provided at Veterans Affairs facilities, with level 1a being the most complex. The facility complexity model includes ICU level, operative complexity level, patient clinical classification, teaching status characteristics, amount of research funding, complex clinical programs provided (eg, invasive catheterization laboratory, neurosurgery, or transplant), rurality, care provided in the community, and mental health programs provided.

Compared with patients with COVID-19 in the ICU treated during periods of low COVID-19 ICU demand (ie, ≤25%), the adjusted HR for all-cause mortality was 0.99 (95% CI, 0.81-1.22; *P* = .93) for patients treated during periods when COVID-19 ICU demand was greater than 25% to 50%, 1.19 (95% CI, 0.95-1.48 *P* = .13) when COVID-19 ICU demand was greater than 50% to 75%, and 1.94 (95% CI, 1.46-2.59; *P* < .001) when COVID-19 ICU demand was greater than 75% to 100%. No statistically significant association between COVID-19 ICU demand and mortality was observed among patients with COVID-19 who were not in the ICU. The eTable in the Supplement provides the adjusted HRs for the 2 measures of COVID-19 ICU strain early in the pandemic (ie, March-May 2020) and later in the pandemic (ie, June-August 2020), and these data are consistent with overall study findings.

## Discussion

In this cohort study of patients with COVID-19 in US VA hospitals, receiving treatment during peak COVID-19 ICU demand, with demand describing the caseload of patients with COVID-19 in the ICU when the patient was treated compared with peak COVID-19 ICU caseload, was consistently and independently associated with COVID-19 ICU mortality. In the extreme case, the adjusted hazard of death was 1.94 for patients with COVID-19 treated in the ICU during periods with greater than 75% to 100% of the peak COVID-19 ICU caseload. The finding that COVID-19 ICU demand was associated with increased mortality for patients with critical COVID-19 early in the pandemic (ie, March-May) and later in the pandemic (ie, June-August) supports the overall study results that suggested that strains on critical care capacity were associated with increased COVID-19 ICU mortality.

Tracking COVID-19 ICU demand may be useful to hospital administrators and health officials as they seek to implement interventions to optimize outcomes for patients with COVID-19.^8^ COVID-19 ICU demand can be calculated only retrospectively (because the peak number of patients with COVID-19 in the ICU can be assessed only retrospectively). However, facilities can identify the peak surge caseload since the pandemic started, in March 2020, and prospectively monitor COVID-19 ICU demand. Facilities within a health care system or within a geographic region could collaborate to triage patients with critical COVID-19 to sites with greater ICU capacity to reduce strain on any 1 facility.^9,10^ Future research is urgently needed to investigate the mechanisms by which COVID-19 ICU demand may be associated with increased mortality; it is imperative that we understand the degree to which patient characteristics (eg, disease severity) or facility issues (eg, staffing) contribute to the association between COVID-19 ICU strain and poor patient outcomes among patients with critical COVID-19.

We did not have a formal measure of ICU capacity, because VA ICU bed availability is not fixed but instead depends on staffing availability; therefore, we calculated COVID-19 ICU load as the ratio of ICU COVID-19 occupancy to the maximum ICU bed number as a surrogate for COVID-19 ICU capacity. Although the association between COVID-19 ICU load and patient mortality was statistically significant, it was neither as consistent over time nor as robust as the association between COVID-19 ICU demand and mortality. We hypothesize that facilities increased their critical care capacity in response to the pandemic and that the degree of this augmentation varied across facilities. Therefore, the comparison with a fixed number of patient beds was likely a relatively poor measure of ICU capacity during the pandemic. Given that hospitals are charged with caring for patients with non–COVID-19 critical illness as well as patients with COVID-19, future studies should seek to examine whether measures of critical care strain that include all patients in the ICU (not just those with COVID-19) are associated with patient outcomes. Future studies should also evaluate whether ICU load provides an adequate measure of strain across the broad spectrum of VA and non-VA hospitals, which vary greatly in prepandemic ICU bed number and the potential to augment capacity during a pandemic. Our overall study findings are supported by cohort studies from 2013^11^ and 2018^12^ demonstrating that as ICUs are strained, mortality increases.^11,12^

It may be the case that during periods of peak ICU caseload, patients who would be admitted to the ICU under more typical conditions are instead admitted to the ward.^13^ Our data did not allow us to examine this issue directly; however, we did examine outcomes associated with COVID-ICU strain separately among patients in the general ward and patients in the ICU. Although the association between COVID-19 ICU load and general ward mortality was statistically significant, it varied over time (ie, early vs later in the pandemic). Future research should examine how critical care strains may be associated with outcomes in the general ward for patients with COVID-19 and those without COVID-19.

### Limitations

This study has several limitations. First, this study evaluated care of patients with COVID-19 at VA hospitals; future studies should examine the association between COVID-19 ICU burden and mortality in non-VA facilities. Second, this study focused on COVID-19 mortality; future studies should examine the potential associations of COVID-19 ICU load and demand with outcomes among patients without COVID-19. Third, the results of this study should not be interpreted as a statement on scarcity of critical care or mechanical ventilation; we have no data to suggest that patients needing critical care or mechanical ventilation did not receive this care.^14^ Fourth, although the risk adjustment models included demographic and clinical characteristics, they did not include social determinants of health (eg, income or education), which may contribute to COVID-19 mortality. Fifth, we did not examine changes in ICU staffing during the study period. Sixth, we do not have a measure of the degree to which facilities expanded ICU capacity during the pandemic. Seventh, patients with COVID-19 who were admitted to the ICU service could have physically been in diverse settings, including locations designated as the COVID-19 ICU, such as surgical ICUs; some patients with critical COVID-19 were cared for by the ICU team but were physically located in the emergency department. Eighth, related to the observed change in mortality over time, our results suggest that changes in patient characteristics and measures of COVID-19 ICU strain were associated with some of the variation in mortality over time; however, given the observational nature of these data, causality cannot be inferred. Other potential causes (eg, use of medications, such as remdesivir and dexamethasone; clinical practices, such as proning; and unmeasured changes in patient characteristics, such as susceptibility) may have contributed to changes in COVID-19 mortality.^15^

## Conclusions

In this cohort study of patients with COVID-19 in US VA hospitals, COVID-19 ICU demand—a measure of COVID-19 ICU caseload when a patient was treated compared with peak COVID-19 ICU caseload—was associated with mortality among patients with COVID-19 in the ICU. Public health officials and hospital administrators may seek to prevent high COVID-19 ICU demand to optimize outcomes for patients with COVID-19.

## Notes

Supplement. **eTable.** Proportional Hazard Results for COVID-19 ICU Strain: Time From Admission to 30 Days Postdischarge or Death by Temporal Period
