# Supplementary material for: Association of Intensive Care Unit Patient Load and Demand With Mortality Rates in US Department of Veterans Affairs Hospitals During the COVID-19 Pandemic
Source: JAMA Netw Open. 2021 Jan 19;4(1):e2034266. doi: 10.1001/jamanetworkopen.2020.34266 (PMC7816100; doi:10.1001/jamanetworkopen.2020.34266)
Supplement: Supplement. — eTable. Proportional Hazard Results for COVID-19 ICU Strain: Time From Admission to 30 Days Postdischarge or Death by Temporal Period [file jamanetwopen-e2034266-s001.pdf]

Supplemental Online Content

Bravata DM, Perkins AJ, Myers LJ, et al. Association of intensive care unit patient load and demand with mortality rates in US Department of Veterans Affairs hospitals during the COVID-19 pandemic. *JAMA Netw Open*. 2021;4(1):e2034266. doi: 10.1001/jamanetworkopen.2020.34266

eTable. Proportional Hazard Results for COVID-19 ICU Strain: Time From Admission to 30 Days Postdischarge or Death by Temporal Period

This supplemental material has been provided by the authors to give readers additional information about their work.

© 2021 Bravata DM et al. *JAMA Network Open.*

eTable. Proportional Hazard Results for COVID-19 ICU Strain: Time From Admission to 30 Days Postdischarge or Death by Temporal Period

Baseline Characteristic	Overall		General Ward Only		Intensive Care Unit (ICU)	
	HR (95% CI)	P-value	HR (95% CI)	P-value	HR (95% CI)	P-value
**March-May 2020***						
**COVID-19 ICU-Load**:† Mean number COVID-19 ICU patients during stay/Number of ICU Beds		0.049		0.010		0.243
≤25% (reference)	1.00		1.00		1.00	
>25% to 50%	1.25 (0.98, 1.59)		1.46 (0.94, 2.26)		1.14 (0.85, 1.53)	
>50% to 75%	0.97 (0.71, 1.34)		0.65 (0.34, 1.23)		1.07 (0.73, 1.56)	
>75% to 100%	1.38 (0.93, 2.05)		1.98 (0.96, 4.10)		1.36 (0.85, 2.19)	
>100%	1.70 (0.95, 3.02)		1.03 (0.26, 4.12)		1.92 (0.99, 3.69)	
**COVID-19 ICU-Demand**:† Mean number COVID-19 ICU patients during stay/Maximum number of COVID patients		<0.001		0.264		<0.001
≤25% (reference)	1.00		1.00		1.00	
>25% to 50%	1.01 (0.80, 1.28)		1.42 (0.96, 2.10)		0.89 (0.66, 1.20)	
>50% to 75%	1.08 (0.84, 1.40)		1.12 (0.71, 1.77)		1.09 (0.80, 1.49)	
>75%	1.71 (1.24, 2.34)		1.13 (0.63, 2.04)		2.18 (1.48, 3.20)	
**June-August 2020***						
**COVID-19 ICU-Load**:† Mean number COVID-19 ICU patients during stay/Number of ICU Beds		0.367		0.047		0.753
≤25% (reference)	1.00		1.00		1.00	
>25% to 50%	1.19 (0.89, 1.59)		0.99 (0.56, 1.76)		1.19 (0.84, 1.68)	
>50% to 75%	2.08 (0.79, 5.47)		4.01 (1.20, 13.39)		0.84 (0.11, 6.65)	
>75% to 100%	--		--		--	
>100%	--		--		--	
**COVID-19 ICU-Demand**:† Mean number COVID-19 ICU patients during stay/Maximum number of COVID patients		0.016		0.381		0.023
≤25% (reference)	1.00		1.00		1.00	
>25% to 50%	1.20 (0.95, 1.52)		1.22 (0.84, 1.77)		1.22 (0.90, 1.66)	
>50% to 75%	1.45 (1.13, 1.87)		1.46 (0.94, 2.26)		1.47 (1.06, 2.02)	
>75%	1.59 (1.12, 2.25)		1.34 (0.72, 2.51)		1.90 (1.23, 2.94)	

*The temporal period refers to the date of the first COVID-19 laboratory test per patient.

© 2021 Bravata DM et al. *JAMA Network Open.*
